# Probing the neurochemical basis of synaesthesia using psychophysics

**DOI:** 10.3389/fnhum.2014.00089

**Published:** 2014-02-20

**Authors:** Devin B. Terhune, Seoho M. Song, Mihaela D. Duta, Roi Cohen Kadosh

**Affiliations:** Department of Experimental Psychology, University of OxfordOxford, UK

**Keywords:** disinhibition, GABA, serotonin, synaesthesia

## Abstract

The neurochemical mechanisms that contribute to synaesthesia are poorly understood, but multiple models implicate serotonin and GABA in the development of this condition. Here we used psychophysical tasks to test the predictions that synaesthetes would display behavioral performance consistent with reduced GABA and elevated serotonin in primary visual cortex. Controls and synaesthetes completed the orientation-specific surround suppression (OSSS) and tilt-after effect (TAE) tasks, previously shown to relate to GABA and serotonin levels, respectively. Controls and synaesthetes did not differ in the performance parameter previously associated with GABA or in the magnitude of the TAE. However, synaesthetes did display lower contrast difference thresholds in the OSSS task than controls when no surround (NS) was present. These results are inconsistent with the hypothesized roles of GABA and serotonin in this condition, but provide preliminary evidence that synaesthetes exhibit enhanced contrast discrimination.

## Introduction

Grapheme-color synaesthesia is an unusual neurological condition in which letters and numerals reliably elicit involuntary color experiences (for a review, see Ward, 2013). Individuals with this condition have been shown to display enhanced color discrimination (Yaro and Ward, 2007; Banissy et al., 2009, 2013) and color working memory (Terhune et al., 2013), which may relate to larger visual-evoked potentials for stimuli that bias the parvocellular visual pathway in this population (Barnett et al., 2008). The extents to which these results are reflective of broader differences in visual processing among synaesthetes remain unknown, but predictions regarding such differences can be derived from competing models of this condition. Two neurotransmitters, γ-aminobutyric acid (GABA) and serotonin (5-HT), have been proposed to contribute to synaesthesia by different models (Grossenbacher and Lovelace, 2001; Cohen Kadosh and Henik, 2007; Brang and Ramachandran, 2008; Brogaard, 2013). Both play fundamental, but differential, roles in visual processing and are strong candidates for exploring the neurochemistry of synaesthesia.

GABA is a major cortical inhibitory neurotransmitter, and GABAergic interneurons comprise approximately 1/6 of neurons in the cortex (Buzsáki et al., 2007). These cells subserve a range of cortical processes such as the segregation of competing neuronal assemblies, cortical maturation, and the shaping of network oscillatory patterns (Möhler, 2007; Buzsáki and Wang, 2012). Resting-state GABA levels in primary visual cortex, as measured by magnetic resonance spectroscopy (MRS; Stagg et al., 2011a; Puts and Edden, 2012), have been shown to covary with orientation discrimination (Edden et al., 2009) and orientation-specific surround suppression (OSSS; Yoon et al., 2010). Specifically, it has been observed that primary visual cortex GABA concentrations are negatively correlated with orientation detection thresholds (Edden et al., 2009) and positively associated with an orientation suppression ratio believed to index inhibition (Yoon et al., 2010). In addition, several studies have demonstrated that GABA_A_ agonists attenuate visual processing along with its electrophysiological correlates (Giersch and Herzog, 2004; Watson et al., 2009; van Loon et al., 2012). It has been argued that disinhibition theories of synaesthesia (Grossenbacher and Lovelace, 2001; Cohen Kadosh and Henik, 2007), which propose that synaesthesia arises from disinhibited feedback from higher cortical areas, predict that synaesthesia is characterized by reduced GABA concentrations in synaesthesia-relevant regions (Hubbard et al., 2011; Specht, 2012). That is, the diminished cortical inhibition proposed to contribute to synaesthetic perception by these theories should be associated with lower GABA levels. A number of studies have provided direct or indirect evidence in support of disinhibition models (e.g., Cohen Kadosh et al., 2009; Terhune et al., 2011), but none have directly evaluated the prediction of reduced GABA in the synaesthetic brain.

Serotonin is a neurotransmitter that plays an instrumental role in a wide range of functions including visual processing. In particular, evidence from the psychopharmacological literature indicates that serotonergic pathways, especially those involving the S2a serotonin receptor, are implicated in visual aberrations produced by amphetaminergic compounds such as methylendioxymethamphetamine (MDMA; Brown et al., 2007; Dickson et al., 2009; White et al., 2013) or hallucinogens such as psilocybin (Marek and Aghajanian, 1998; Kometer et al., 2011, 2013). Perhaps unsurprisingly, serotonergic neurons have also been suggested to regulate visual-motor gating (Pum et al., 2008) as well as cross-modal/cortical sensory integration (Jitsuki et al., 2011; Takahashi, 2011). To date, two hypotheses have highlighted serotonin’s role in the development of synaesthesia (Brang and Ramachandran, 2008; Brogaard, 2013). Brang and Ramachandran (2008) have argued that the S2a serotonin receptor may underlie hyperconnectivity between the fusiform gyrus and area V4, which has been predicted to play an instrumental role in the development of this condition (Hubbard, 2007; Hubbard et al., 2011). Brogaard (2013) similarly argues that elevated serotonin in the striate cortex may contribute to the development of synaesthesia and may underlie acquired and induced synaesthesias. Perhaps the best evidence to date for these models comes from a recent meta-analytic review, which shows that the induction of synaesthesia-like experiences in non-synaesthetes is most reliably observed with serotonin agonists, such as lysergic acid diethylamide (LSD), mescaline, and psilocybin (Luke and Terhune, 2013). There is also preliminary evidence that serotonin agonists enhance synaesthesia in congenital synaesthetes (Simpson and Mckellar, 1955; Luke et al., 2012). However, these studies suffer from a number of methodological limitations (Luke and Terhune, 2013) and only provide preliminary support for serotonin models. Although congenital and induced synaesthesias may not share the same neurochemical mechanisms, these studies do suggest that serotonin may be implicated in synaesthesia.

The aim of the present study was to test predictions derived from disinhibition and serotonin models of synaesthesia using proxy psychophysics measures of GABA and serotonin. Toward this end, controls and synaesthetes completed a task measuring OSSS (Xing and Heeger, 2001; Zenger-Landolt and Heeger, 2003; Yoon et al., 2009, 2010). Target identification is poorest when the surrounding region has an orientation parallel to the target and performance is facilitated by surround suppression (Chubb et al., 1989; Xing and Heeger, 2001; Yoon et al., 2009). The magnitude of this suppression effect has been long believed to be driven by inhibitory mechanisms and has recently been shown to correlate positively with GABA concentrations in primary visual cortex (Yoon et al., 2010). Insofar as disinhibition theories predict reduced GABA in synaesthesia, we tested the prediction that synaesthetes would display less surround suppression owing to a lower inhibition-derived sharpening of the stimulus orientation. In particular, we expected that synaesthetes would perform less optimally than controls where inhibitory fine-tuning processes would benefit the most such as when the parallel-oriented surround obscures the target.

Measurement of serotonin-related performance relied on a visual phenomenon known as the *tilt-after effect* (TAE; Paradiso et al., 1989; Masini et al., 1990; Murray et al., 2012). When participants are adapted to a single stimulus with a certain angular orientation, their perception of the following stimuli’s angular orientation tends to be biased in the direction opposite that of the adaptive stimulus (Masini et al., 1990). This is due to the saturation of neurons that are specific to the orientation of the adaptive stimulus, which dampens the neurons’ sensitivities to subsequent stimuli (He and Macleod, 2001; Murray et al., 2012). Decreasing cortical serotonin, such as through tryptophan depletion, hinders this inhibition, stretches the neurons’ tuning bandwidth, and augments the magnitude of the TAE (Masini et al., 1990; Brown et al., 2007; Murray et al., 2012). If synaesthesia is characterized by elevated serotonin in primary visual cortex, synaesthetes should display an attenuated TAE.

## Methods

### Participants

Sixteen controls (14 female, *M*_Age_ = 23.1, *SD* = 4.3) and 15 grapheme-color synaesthetes (13 female; *M*_Age_ = 25.1, *SD* = 4.8), all of whom were right-handed and had normal or corrected-to-normal vision, provided informed consent to participate in this study in accordance with approval from a local ethics committee. The two groups did not differ in gender distributions, Fisher’s exact *p* = 1, age, *F* < 1.5, or years of (post-secondary) education (controls: 3.8 ± 1.9, synaesthetes, 4.5 ± 2.6), *F* < 1.

In order to compute consistency of grapheme-color associations, participants selected colors using a color picker for the numbers 0 through 9 in random order three times in a serial fashion (Eagleman et al., 2007; Rothen et al., 2013). On the second and third rounds, controls were instructed to try to select the same color that they previously selected for each respective grapheme. Synaesthetes displayed greater consistency (lower values reflect greater consistency) with a measure based on city block distances in RGB color space (Eagleman et al., 2007) (controls: 1.85 ± 0.22; synaesthetes: 0.58 ± 0.04; *H* = 13.81, *p* < 0.001, $\eta_{p}^{2}=0.51$ [CIs: 0.23, 0.66]) and a measure based on Euclidean distances in CIELUV color space (Rothen et al., 2013) (controls: 143.91 ± 14.58; synaesthetes: 33.03 ± 15.06; *H* = 16.58, *p* < 0.001, $\eta_{p}^{2}=0.49$ [CIs: 0.21, 0.65]).

### Materials

#### Orientation-specific surround suppression (OSSS)

**Figure 1:**
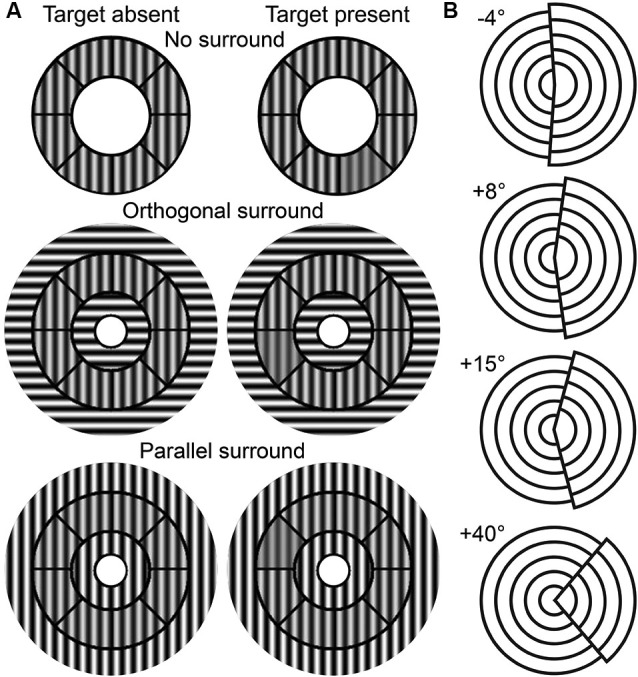
**Task stimuli**. **(A)** OSSS task stimuli as a function of target presence and surround condition. **(B)** TAE task stimuli examples.

The OSSS task measures the participant’s ability to discriminate contrast differences between targets in variously oriented surrounds (Zenger-Landolt and Heeger, 2003). Stimuli were roundels that contained contrast reversing (4 Hz), gray-scale sinusoidal gratings with a spatial frequency of 2.2 cycles per degree (see Figure 1A). Each stimulus contained an *annulus* that was divided into eight sectors and the *surround* that was partitioned into central and outer regions. The central region extended from the center of the stimulus to the inner radius of the annulus (2.9°). The outer region extended from the outer radius of the annulus (5.6°) to an eccentricity of 9.2°. The contrast of both surround regions was kept at 100%. The central portion of the surround included a circle of radius 1.0°.

The OSSS task had three surround conditions: *no surround* (NS), *parallel surround* (PS), and *orthogonal surround* (OS). The PS and OS conditions contained gratings in the surround that were parallel or perpendicular to the annulus, respectively. 50% of the trials randomly included a target (a single randomly selected annular sector with a lower contrast relative to the remainder of the annulus sectors) and participants judged whether the target was present or absent. The contrast of the target ranged from 38% to 73% and was parametrically varied in steps of 5% depending on performance using a 3-up, 1-down adaptive staircase procedure. The contrast of the non-target annular regions was fixed at 75%. Stimuli were presented for 750 ms with an interstimulus interval of 1050 ms.

#### Tilt-after effect (TAE)

This task provides a measure of the TAE (Paradiso et al., 1989; Masini et al., 1990). The stimuli consisted of two offset, 4-layered circular sectors (see Figure 1B). The right-hand sector had a larger diameter than the left, and collectively, the stimulus had a diameter of 9°. The right sector was tilted by a certain angle toward the left (*negative* tilt) or the right (*positive* tilt) of the vertical midline. There were three phases in this task: *pre-adaptation*, *adaptation*, and *post-adaptation*. In the pre-adaptation phase, participants were presented with stimuli that contained one of nine randomly-selected tilt angles (−4° to +4° with 1° intervals), and judged whether the tilt was negative (leftward) or positive (rightward). During the adaptation phase, participants were exposed to a stimulus with either a +15° or a +40° tilt angle for 90 s. Participants were instructed to scan over the stimulus without fixating on a single position in order to avoid after-images. In the post-adaptation phase, participants judged the tilt direction of stimuli of thirteen possible tilt angles (−4° to +8° with 1° intervals). Stimuli were presented in 10-trial blocks that were interspersed with a 5 s adaptation interval. Stimuli in the pre- and post- adaptation phases were presented for 100 ms with interstimulus intervals of 500 ms.

### Procedure

Participants were recruited for a research study on synaesthesia (synaesthetes) or visual perception (controls) through advertisements at the University of Oxford. Participants completed the tasks in counterbalanced order on a Dell P190ST monitor (resolution: 1280 × 1024; refresh rate: 60 Hz) at a distance of 50 cm. The OSSS was implemented using the Psychophysics Toolbox software^1^ (Brainard, 1997; Kleiner et al., 2007) for MATLAB® (2012a, MathWorks Inc, Natwick MA), whereas stimulus presentation for the TAE task was implemented using E-Prime® (2.0, Psychology Software Tools Inc., Sharpsburg PA). Responses were recorded using a Cedrus® response pad (Cedrus Corporation, San Diego, CA). Participants completed a practice block of 20 trials in the OSSS task and one 150-trial block of the NS, OS, and PS conditions with the OS and PS block order counterbalanced. In the TAE task, participants completed 90 trials in the pre-adaptation phase and 26 10-trial blocks of each tilt angle in the post-adaptation phase with tilt angle conditions counterbalanced.

### Data analysis

Data in the OSSS task were modeled using the Palamedes Toolbox (Prins and Kingdom, 2009) in MATLAB. The probabilities of a correct response to targets at each contrast level were fitted with a Weibull function defined by four parameters: threshold α, slope β, guess rate γ, and lapse rate λ. Threshold and slope were set as free parameters that were estimated using maximum likelihood estimation, whereas guess and lapse rates were fixed at 0 and 0.1, respectively. In addition to the loss of one synaesthete’s data due to a technical error, nine participants’ data displayed poor model fit (*p*Devs < 0.05; Kingdom and Prins, 2010) in one or more conditions. Model fit was substantially improved through the removal of a single outlier in seven participants (two controls, five synaesthetes), whereas the data of two participants (one control, one synaesthete) could not be improved and thus were excluded from the analyses (the principal results [including the Group differences] are the same if these participants are included). The analyses of the OSSS data therefore included 15 controls and 13 synaesthetes. Model fit did not differ as a function of Condition or Group, nor was there an interaction, *F*s < 1, *p*s > 0.34. The 79% threshold of the psychometric function (*contrast difference threshold*) at each condition was used as our principal dependent measure of interest (Yoon et al., 2010). Threshold values provide a measure of the discriminability of the stimulus with lower values (in the present study) reflecting superior discrimination. As a secondary measure of interest, we also analysed function slopes, which index the steepness of the psychometric function fit to the data. More negative slopes indicate greater steepness of the function and thus greater deterioration in performance from one contrast difference to the next.

The same procedure was applied to the data from the TAE task with the following differences. One synaesthete did not perform the task correctly and provided unusable data and a second synaesthete’s data were lost due to a technical error. Pre-adaptation data in the 15° and 40° conditions did not differ and thus were combined. The probabilities of a positive (rightward) response at each tilt angle were fitted with a logistic function with the same parameter constraints as in the OSSS task. The intersection of the function and the 0.5 threshold was taken as the point of subjective equality (PSE), which corresponds to the tilt angle that is perceived to be approximately equally likely to be negative (leftward) and positive (rightward). Six participants’ data displayed poor model fit (*p*Devs < 0.05); of these, model fit was substantially improved through the exclusion of a single outlier in three participants (two controls, one synaesthete), whereas the data of three participants (two controls, one synaesthete) could not be improved and thus were excluded from the analyses. Accordingly, the analysis of the TAE data included 14 controls and 12 synaesthetes. Model fit did not differ as a function of Condition or Group, nor was there an interaction, *F*s < 2.2, *p*s > 0.13. PSEs for the different conditions were used as the principal dependent measure and function slopes were included as a secondary measure of potential interest. The TAE was quantified as the difference between PSEs before and after adaptation for both the 15° and 40° conditions.

### Statistical analyses

Statistical analyses of the data were conducted using SPSS® (21, IBM) and MATLAB. Outliers were detected using the adjusted boxplot rule (Pernet et al., 2013) and replaced using a nearest neighbor correction (nearest extreme value ±1). Data were analysed with analyses of variance (ANOVA) with Group as the between-groups factor and Surround type (NS v. OS v. PS; OSSS task), or Stimulus type (15° vs. 40° TAE task) as within-groups factors, depending on the analysis. We used unequal variance *t*-tests (Welch, 1947) when data violated the assumption of homogeneity of variance across groups. We applied the Greenhouse-Geisser correction when the assumption of sphericity was violated. Uncorrected *df* s are reported for the latter two analyses. To control for the possibility of false positives, a false discovery rate (FDR) correction (Benjamini et al., 2001) was applied to the entire set of *p*-values comprising analyses for both tasks; only corrected *p*-values are reported.

## Results

### Orientation-specific surround suppression (OSSS)

**Figure 2:**
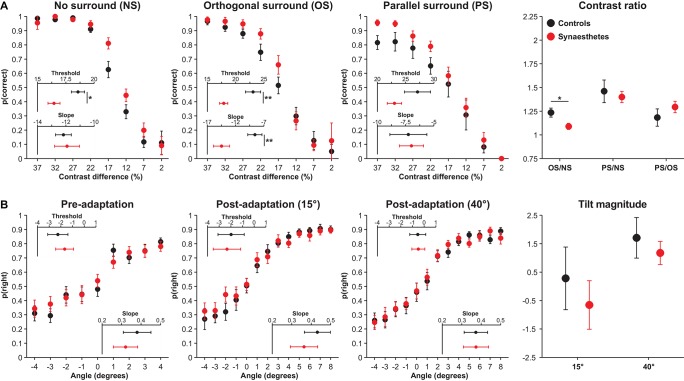
**OSSS (A) and TAE (B) task performance in controls (black) and synaesthetes (red)**. **(A)** Accuracy in the OSSS task as a function of target contrast difference in the different surround conditions and ratios. **(B)** Proportion of right responses as a function of stimulus angle in the different adaptation conditions and tilt magnitudes. Error bars represent 1 *SE*. * *p* < 0.05 **p < 0.01

Performance on the OSSS task is illustrated in Figure 2A. The analysis of contrast difference thresholds revealed main effects of Surround type, *F*_(2,52)_ = 26.34, *p* = 0.013, $\eta_{p}^{2}=0.50$ (CIs: 0.29, 0.63), reflecting a linear increase in thresholds across conditions, and Group, *F*_(1,26)_ = 6.89, *p* = 0.039, $\eta_{p}^{2}=0.21$ (CIs: 0.01, 0.44), reflecting lower contrast difference thresholds (better discrimination) among synaesthetes, but no interaction, *F*_(2,52)_ = 1.09, *p* = 0.49, $\eta_{p}^{2}=0.04$ (CIs: 0.00, 0.16). To determine the breadth of group differences across conditions, particularly since the PS condition requires greater inhibition, we conducted exploratory analyses comparing the groups in the three surround conditions. Synaesthetes displayed lower contrast difference thresholds in the NS, *F*_(1,26)_ = 7.07, *p* = 0.039, $\eta_{p}^{2}=0.21$ (CIs: 0.01, 0.44), and OS, *F*_(1,26)_ = 10.64, *p* = 0.021, $\eta_{p}^{2}=0.29$ (CIs: 0.04, 0.51), conditions, but not in the PS condition, *t*_(26)_ = 1.55, *p* = 0.28, $\eta_{p}^{2}=0.08$ (CIs: 0.00, 0.31). The former two effects remained significant when model fit in the respective condition was included as a covariate, NS: *F*_(1,25)_ = 6.62, *p* = 0.041, $\eta_{p}^{2}=0.21$ (CIs: 0.01, 0.44), OS: *F*_(1,25)_ = 10.70, *p* = 0.021, $\eta_{p}^{2}=0.30$ (CIs: 0.04, 0.52). These results suggest that synaesthetes display superior contrast discrimination than controls.

In the analysis of slopes, there were main effects of Surround Type, *F*_(2,52)_ = 13.34, *p* = 0.013, $\eta_{p}^{2}=0.34$ (CIs: 0.13, 0.49), reflecting a linear decrease in slopes across the three conditions, no effect of Group, *F*_(1,26)_ = 2.24, *p* = 0.28, $\eta_{p}^{2}=0.08$ (CIs: 0.00, 0.30), but a significant interaction, *F*_(2,52)_ = 7.07, *p* = 0.039, $\eta_{p}^{2}=0.21$ (CIs: 0.04, 0.37). Subsidiary analyses revealed that synaesthetes displayed steeper slopes in the OS condition than controls, *F*_(1,26)_ = 9.73, *p* = 0.022, $\eta_{p}^{2}=0.27$ (CIs: 0.03, 0.49), but that the two groups did not differ in the NS, *F*_(1,26)_ = 0.06, *p* = 0.83, $\eta_{p}^{2}<0.01$ (CIs: 0.00, 0.08), or in the PS, *F*_(1,26)_ = 0.02, *p* = 0.89, $\eta_{p}^{2}<0.01$ (CIs: 0.00, 0.03), conditions. The results indicate that synaesthetes exhibited steeper slopes in the OS condition than controls; this appears to have resulted from the fact that synaesthetes exhibited superior discrimination in this condition than controls at contrast differences of 22% and 17%, but thereafter exhibited a greater decline in discrimination relative to controls, resulting in numerically poorer performance at 12% contrast difference.

Insofar as controls and synaesthetes differed in both OS thresholds and slopes and these two variables were strong correlated, *r* = 0.70, *p* < 0.001, we performed two ANCOVAs repeating these analyses controlling for the other variable. Neither analysis was significant, threshold (controlling for slope): *F*_(1,25)_ = 2.22, *p* = 0.28, $\eta_{p}^{2}=0.08$ (CIs: 0.00, 0.31), slope (controlling for threshold): *F*_(1,25)_ = 1.54, *p* = 0.38, $\eta_{p}^{2}=0.06$ (CIs: 0.00, 0.28), indicating a clear interdependence between these two variables. In contrast, the Group difference in NS thresholds remained significant when controlling for slopes, *F*_(1,25)_ = 7.79, *p* = 0.039, $\eta_{p}^{2}=0.24$ (CIs: 0.02, 0.47).

The contrast thresholds for the OS and PS conditions were further evaluated by controlling for performance in the NS condition through the computation of ratios of contrast thresholds. These analyses were performed to examine performance in suppression conditions (OS and PS) controlling for baseline contrast discrimination. Synaesthetes displayed a lower OS/NS ratio, *t*_(26)_ = 2.52, *p* = 0.044, $\eta_{p}^{2}=0.19$ (CIs: 0.00, 0.43), but did not differ in the PS/NS ratio, *t*_(26)_ = 0.46, *p* = 0.73, $\eta_{p}^{2}=0.01$ (CIs: 0.00, 0.16). The former result suggests that synaesthetes display enhanced surround suppression, although this effect became non-significant when controlling for OS slopes, *F*_(1,25)_ = 0.98, *p* = 0.33, $\eta_{p}^{2}=0.04$ (CIs: 0.00, 0.24). The most crucial analysis concerned the PS/OS ratio, which has been associated with GABA concentrations in primary visual cortex (Yoon et al., 2010). If this relationship holds and synaesthetes display reduced GABA in this region, synaesthetes should exhibit a larger ratio. A non-significant Group effect, *F*_(1,26)_ = 0.97, *p* = 0.49, $\eta_{p}^{2}=0.04$ (CIs: 0.00, 0.24), including when controlling for OS slopes, *F*_(1,26)_ = 0.03, *p* = 0.88, $\eta_{p}^{2}<0.01$ (CIs: 0.00, 0.04), shows that this prediction was not supported. Cumulatively, these results suggest that, in contrast with the predictions of disinhibition models, synaesthetes do not display poorer inhibition than controls, but that synaesthetes display superior contrast discrimination when no surround is present.

### Tilt-after effect (TAE)

The results of the TAE are presented in Figure 2B. On the basis of the finding that lower V1 serotonin levels are associated with a more pronounced TAE (Murray et al., 2012), we expected that synaesthetes would display an attenuated TAE relative to controls. First, the two groups did not exhibit differential PSEs in the pre-adaptation phase of the task, *F*_(1,24)_ = 0.25, *p* = 0.73, $\eta_{p}^{2}=0.01$ (CIs: 0.00, 0.19). The analysis of PSEs in the TAE task revealed a main effect of Stimulus type, *F*_(1,24)_ = 9.07, *p* = 0.028, $\eta_{p}^{2}=0.27$ (CIs: 0.03, 0.50), in which adaptation to 40° produced a positive tilt magnitude and adaptation to 15° resulted in a negative tilt, but there was neither a main effect of Group, *F*_(1,24)_ = 0.49, *p* = 0.65, $\eta_{p}^{2}=0.02$ (CIs: 0.00, 0.21), nor an interaction, *F*_(1,24)_ = 0.14, *p* = 0.76, $\eta_{p}^{2}<0.01$ (CIs: 0.00, 0.16). Contrary to earlier findings (Paradiso et al., 1989; Murray et al., 2012), the tilt magnitude at 15° suggested increased rightward responses following adaptation, yet this was unrelated to between-group differences. The analysis of slopes similarly found no pre-adaptation differences as a function of Group, *F*_(1,24)_ = 0.38, *p* = 0.69, $\eta_{p}^{2}=0.02$ (CIs: 0.00, 0.20), main effects of Stimulus type, *F*_(1,24)_ = 0.91, *p* = 0.49, $\eta_{p}^{2}=0.04$ (CIs: 0.00, 0.25), Group, *F*_(1,24)_ = 0.24, *p* = 0.73, $\eta_{p}^{2}=0.01$ (CIs: 0.00, 0.18), or an interaction, *F*_(1,24)_ = 1.84, *p* = 0.33, $\eta_{p}^{2}=0.07$ (CIs: 0.00, 0.30). Cumulatively, these results indicate that controls and synaesthetes do not differ in tilt perception or in the magnitude of the TAE.

## Discussion

This study tested predictions pertaining to altered neurochemistry in synaesthesia derived from disinhibition and serotonin models of this condition using proxy psychophysical measures. In contrast to both models, the two groups did not display differential response patterns on task measures previously associated with visual cortex GABA and serotonin levels. Exploratory analyses revealed that synaesthetes displayed lower contrast difference thresholds in one condition of an orientation task than controls, suggesting superior contrast discrimination. These results are somewhat equivocal with regard to their implications for the involvement of GABAergic disinhibition or elevated serotonin as chemical underpinnings of synaesthesia, but suggest that these neurochemicals are not altered in this condition.

In contrast to the results suggesting enhanced contrast discrimination in synaesthetes relative to controls (see below), the two groups did not differ in the ratio of contrast thresholds in the parallel and OS conditions (PS/OS ratio). The between-groups factor of Group explained ~4% (CIs: 0.00, 0.24; controlling for slope: <0.01 [CIs: 0.00, 0.04]) of the variance in this ratio and therefore, when considering the sample sizes of controls and synaesthetes, renders it unlikely that this null result is due to low statistical power. The PS/OS ratio has previously been shown to correlate with GABA concentrations in primary visual cortex (Yoon et al., 2010) and thus was used to test the prediction, derived from disinhibition models (Cohen Kadosh and Henik, 2007; see also Hubbard et al., 2011), that synaesthetes would exhibit reduced GABA. These results suggest that synaesthetes do not display reduced inhibition in primary visual cortex, but caution should be exerted in the interpretation of these results for multiple reasons.

First, the relationship between GABA and the PS/OS ratio is correlational and this ratio may not provide a robust proxy measure of visual cortex GABA. That is, it may be the case that this measure does not constitute a sufficiently stringent test of the prediction of reduced GABA in synaesthesia. For example, one reason to doubt the link between GABA and PS/OS ratios in the Yoon et al. (2010) study is that patients with schizophrenia were medicated, which may represent an important confound in the measurement of GABA levels in this population (Kegeles et al., 2012). However, it is readily evident that the relationship between GABA and the PS/OS ratio in Yoon et al. (2010) is driven by controls, rather than schizophrenics. Moreover, a recent study found that when applied to the primary visual cortex, anodal transcranial direct current stimulation, previously shown to reduce cortical GABA (Stagg et al., 2009), attenuated surround suppression on a similar orientation discrimination task (Spiegel et al., 2012). This strengthens the claim that surround suppression is indeed related to individual differences in visual cortex GABA.

Second, the evidence for the PS/OS ratio as a proxy measure of GABA in striate cortex comes from a study showing that it correlates with GABA in primary visual cortex, as measured by MRS (Yoon et al., 2010). The available evidence suggests that MRS-derived GABA concentrations seem to reflect extra-synaptic GABA (GABA tone) (Stagg et al., 2011a, b). Accordingly, it is possible that disinhibition-specific differences associated with GABAergic interneurons contributing to the expression of synaesthesia may not be detectable with MRS-derived measures of cortical GABA concentrations. Rather, it may be more informative to focus on the density and distribution of various types of GABA receptors, which are the principle foundations upon which GABA exercises its influences on neuronal activity.

Finally, if we assume that the PS/OS ratio provides a reliable measure of visual cortex GABA, it is plausible that differences in GABAergic activity between controls and synaesthetes are actually restricted to fusiform gyrus or V4 or higher cortical areas such as parietal cortex (e.g., van Leeuwen et al., 2011), rather than primary visual cortex. Indeed, elsewhere we have argued that cortical hyperexcitability in primary visual cortex in synaesthetes does not play a causal role in the online experience of synaesthesia; rather, it may have only contributed to the expression of synaesthesia at an early developmental stage (Terhune et al., 2011). At present, we are unable to discriminate between these competing interpretations of the PS/OS results. Nevertheless, the results clearly do not support disinhibition theories, but the extent to which they are inconsistent with them is as yet unclear.

Controls and synaesthetes also completed the TAE task, which provided a measure of the magnitude of the TAE. The TAE is believed to be augmented in conditions characterized by low serotonin in primary visual cortex (Paradiso et al., 1989; Brown et al., 2007; Murray et al., 2012) and was used to test the prediction that synaesthetes would display a reduced TAE. In contrast with this prediction, the two groups did not differ in the magnitude of the TAE, suggesting that synaesthesia is not characterized by elevated serotonin in striate cortex. As was the case with the critical parameter in the OSSS task (PS/OS ratio), the magnitude of this effect was very small (~2%; CIs: 0.00, 0.21) and thus, given our sample sizes, it is unlikely that this analysis was underpowered. As is the case with the OSSS task, there are a number of explanations for these null results. First, previous research used the TAE to identify serotonin deficiencies (Masini et al., 1990; Brown et al., 2007; Murray et al., 2012) and thus it could be argued that the TAE is not well suited to detect elevated serotonin levels. We failed to observe any clear evidence for ceiling effects on this task and thus this explanation seems unlikely. A second possibility is that the TAE is not a reliable measure of visual cortex serotonin levels. Given the number of studies linking serotonin with the TAE, we also find this unlikely. For example, one study found that acute tryptophan depletion augmented the magnitude of the TAE (Masini et al., 1990). Tryptophan is the physiological precursor to serotonin and has been shown to produce a temporary reduction in 5-HT (2) receptor binding (Yatham et al., 2001). Accordingly, we interpret the current results to suggest that controls and synaesthetes do not differ in V1 serotonin. If serotonin plays a decisive role in the occurrence of synaesthesia, the present results suggest that it is most likely in other (downstream) cortical regions, such as fusiform gyrus and V4.

Recent evidence has surfaced raising concerns about the brain region responsible for the TAE. In particular, it has been suggested that the TAE is driven by retinotopic as well as cortical mechanisms (Knapen et al., 2010; Mathot and Theeuwes, 2013). This possibility is controversial because a retina-based mechanism would be unable to account for differences in the TAE caused by ecstasy consumption (Brown et al., 2007; Murray et al., 2012) or by tryptophan depletion (Masini et al., 1990). The former studies arguably challenge the validity of the TAE as an indirect measure of cortical serotonin. However, the psychophysical paradigms used to demonstrate a retinotopic component to the TAE are only conceptually similar to the assays used in the present study (e.g., they employ structurally disparate stimuli). Hence, this limitation of the TAE does not severely undermine the TAE’s capacity to represent cortical serotonin.

Further work is required to explore whether GABA and serotonin are implicated in synaesthesia. This may be achieved by using MRS to measure GABA in fusiform gyrus and V4 or through methods for modulating visual cortex serotonin (e.g., tryptophan depletion). The study of synaesthesia-like experiences following the intake of serotonin agonists has especially strong potential to inform our understanding of the role of this neurochemical in synaesthesia (Brang and Ramachandran, 2008; Brogaard, 2013; Luke and Terhune, 2013). There is also considerable potential that disinhibition and serotonin models can be integrated. For instance, it has been shown that elevated serotonin shifts the balance between excitation and inhibition in favor of excitation (Moreau et al., 2010). Accordingly, elevated serotonin could potentially give rise to a state of cortical hyperexcitability and attenuated inhibition (see also Terhune et al., 2011).

A novel finding of this study is that synaesthetes displayed superior contrast difference thresholds than controls. This effect was present both in the NS and OS conditions and thus is unlikely to reflect a difference in surround suppression, although only the former difference remained when controlling for condition slopes. Synaesthetes similarly exhibited steeper slopes in the OS condition, which reflect the steepness of the psychometric function fit to individual participants’ data. Although synaesthetes outperformed controls at mid-range contrast differences (17 and 22%), their performance was more greatly taxed at lower contrast differences (12 and 7%); below we speculate as to why this might be. Insofar as OS thresholds and slopes were inter-dependent, the apparent superior contrast discrimination among synaesthetes in the OS condition should be interpreted with caution. Superior performance among synaesthetes in the NS condition is suggestive of superior contrast discrimination in this population, but further research is necessary to determine the replicability of this effect. The observed performance difference appears to converge nicely with those of Barnett et al. (2008) who observed that synaesthetes display enhanced visual-evoked potentials for stimuli that are preferentially processed by the parvocellular visual pathway but not those that bias the magnocellular pathway. Although the low spatial frequency of the stimuli used in the OSSS task (see Section Methods) biases magnocellular neurons (e.g., Derrington and Lennie, 1984), the observed group difference pertains to the detection of contrast differences between the target and the surrounding sectors of the annulus. That is, the target differed in contrast from the surrounding sections, but not in spatial frequency. The magnocellular pathway saturates at low levels of contrast (Merigan and Maunsell, 1993; Lee, 1996) whereas the parvocellular system is recruited for ~10% contrast and greater (Tootell et al., 1988). Accordingly, it is significant that the group differences in the NS condition of the OSSS task, based on the absence of overlap of standard error bars (see Figure 2A), are only present at 12% and higher contrast differences. This suggests that contrast discrimination advantages among synaesthetes emerge at contrasts for which the parvocellular pathway is preferentially recruited; these results are therefore consistent with the proposal that synaesthetes exhibit enhanced responsiveness of the parvocellular system (Barnett et al., 2008; see also Rothen et al., 2012).

The observations that synaesthetes displayed selectively superior contrast discrimination, but did not differ in PS/OS ratios or the TAE (see below), are also significant for multiple reasons. First, as has been noted elsewhere (Gross et al., 2011; Radvansky et al., 2011; Terhune et al., 2013), the specificity of performance advantages reduces the likelihood that superior performance is driven by greater motivation among synaesthetes (see also Banissy et al., 2013). Second, it is noteworthy that synaesthetes no longer outperformed controls in the OSSS condition that most strongly taxes cortical inhibition (PS). It is plausible that superior contrast discrimination and deficient cortical inhibition come into conflict with one another in this condition and that elevated contrast discrimination among synaesthetes may mask inhibition differences. Further research is required to more precisely investigate this possibility.

The present results should be interpreted within the context of the limitations of this study. We have argued that the analyses showing null results pertaining to the psychophysical parameters putatively related to GABA and serotonin are unlikely to be underpowered given the small effect sizes. We have also corrected for multiple analyses to reduce the likelihood of reporting false positive results. However, as in any study with small samples, it is possible that the observed effects will disappear with larger sample sizes. It is plausible that the differential performance patterns of controls and synaesthetes are due to elevated motivation in the latter group rather than differences in contrast discrimination, but we have argued that this is unlikely. Finally, the fits of psychometric functions to behavioral data were poor for a subset of participants, who displayed poor performance in one or more contrast differences (OSSS task) or angles (TAE task). We were able to correct most instances of poor fit with outlier removal, but this might reduce the generalizability of our results. Taken together, these limitations warrant that the contrast discrimination difference between controls and synaesthetes be replicated before firm conclusions regarding enhanced contrast discrimination among synaesthetes can be advanced.

### Conclusion

This study contrasted controls and synaesthetes in two psychophysical measures in order to test predictions derived from disinhibition and serotonin models of synaesthesia. The two groups did not differ in the performance patterns predicted by these models, but synaesthetes displayed superior contrast discrimination than controls in the absence of surround stimuli. Although the tasks used do not constitute rigorous evidence against these models, they suggest that synaesthetes do not exhibit atypical GABA or serotonin levels in primary visual cortex.

## Conflict of interest statement

Conflict of Interest Statement: The authors declare that the research was conducted in the absence of any commercial or financial relationships that could be construed as a potential conflict of interest.

## Footnotes

^1^http://psychtoolbox.org
